# Implications of subtalar joint motion for muscle and joint loading during running

**DOI:** 10.1186/1757-1146-5-S1-O45

**Published:** 2012-04-10

**Authors:** Uwe G Kersting

**Affiliations:** 1 Center for Sensory-Motor Interaction, Department of Health Science and Technology, Aalborg University, 9220 Aalborg, Denmark

## Background

Rearfoot pronation is one of the factors having been linked to overuse injuries in running (1). Many studies have used shoe eversion as a measure for movement about the subtalar joint axis. Others used foot movement expressed as rotations about the anatomical main axes (2). If joint coupling and with that muscle loading is to be investigated it may be beneficial to assess rotations about the anatomical axes, namely the talocrural and subtalar joints.

The aim of this study was to describe how the relationship of the two ankle joint movements is affected by moderate geometric changes of the midsole of a running shoe. Secondly, the relationship of total ankle movement in main anatomical planes and the two anatomical ankle joints was to be explored.

## Materials and methods

Eleven experienced runners were asked to run in a running shoe (Nike Pegasus) with neutral (NE), +4° varus (VR) and –4° valgus (VG) midsole. Subjects were given 1 – 2 km of running to accommodate. Three-dimensional kinematics were recorded using an eight-camera system (Motion Analysis Corp., Eva, 120 Hz). Ground reaction forces were sampled at 1200 Hz from 2 force plates (Bertec).

A lower extremity model was scaled to each individual and inverse dynamics analysis carried out in the Any Body Modelling System (3). Subtalar and talocrural joint kinematics, joint kinetics and muscle activations were extracted after optimisation. Additionally, foot rotations with respect to the leg segment in the cardinal anatomical planes were extracted for comparison. Regression techniques were employed to test for relationships of kinematics and muscle activations (p<.05).

## Results

Sagittal plane ankle movement consistently showed significant correlations with talocrural joint excursions while movement about the subtalar joint showed moderate to high correlations to movement in the frontal and transverse planes. However, for some subjects these relationships were inverted for non-neutral shoe conditions. Muscle forces were generally closer related to rotations about the anatomical joint axes than projected to the cardinal planes.

## Conclusions

When addressing the relationship between soft tissue loading and joint kinematics anatomical joint axes orientations should be preferred. It remains, however, open to verification how such loading distribution is affected by anatomical variations in individuals.
